# Gender Differences in the Propensity to Start Gambling

**DOI:** 10.1007/s10899-023-10232-z

**Published:** 2023-07-04

**Authors:** Alejandro Díaz, Jaume García, Levi Pérez

**Affiliations:** 1 Department of Economics, University of Oviedo, Gijón, Spain; 2 Department of Economics and Business, Universitat Pompeu Fabra, Barcelona, Spain

**Keywords:** Gender differences, Gambling, Propensity, Survival analysis

## Abstract

Gambling opportunities have greatly expanded in recent years leading to an alternative form of leisure but also raising social concerns. Participation in such activities may be conditioned by individual characteristics affecting the willingness of individuals to gamble, including gender, but also by time effects linked to the availability and exposure of gambling. Using data from Spain, estimates from a time-varying split population duration model show significant gender differences in the propensity to start gambling (men’s episodes as non-gamblers were observed to be shorter than women’s). Additionally, expansion of gambling opportunities over time is found to be correlated with an increase in the propensity to start gambling. Both men and women are clearly more likely to start gambling at earlier ages than before. These results are expected to improve knowledge of gender differences in terms of consumer decision making about gambling and to be helpful in designing public policies for gambling.

## Introduction

Recently, the prevalence and scale of gambling has increased significantly, becoming an alternative form of leisure and entertainment within an increasingly crowded and highly competitive supply. This trend has been accompanied by a growth in both gambling participation and expenditure (Abbott et al., 2014). As the expansion of gambling opportunities continues, there is however considerable public controversy over social concerns, and potential harm and gambling-related disorders. Opponents to gambling usually base their objections on concerns such as the regressivity of gambling taxation, meaning that gambling revenues are disproportionately drawn from low-income people (Gandullia & Leporatti, 2018; Perez & Humphreys, 2011); moral consideration (Basham & White, 2002); and gambling-related harms beyond the loss of money (e.g., addiction, crime, work performance, social life issues…) (Delfabbro & King, 2019).

In fact, governments worldwide hold divergent positions on gambling. While some countries have actually banned all forms of gambling, many others allow a wide range of quite different gambling activities. Where gambling is allowed, governments have traded its negative aspects for the potential benefits—mostly economic—of regulating and taxing it. These different gambling regimes are usually determined by cultural and/or religious reasons (e.g., Islamic laws do not allow for any form of gambling) and by regional and historical market factors. Market availability of gambling products is indeed determined, among other factors, by regulatory issues that may in some way affect consumer exposure and attitudes toward them.

It is actually expected that the odds of starting gambling may be conditioned by the availability and exposure of gambling opportunities, which are undoubtedly conditioned by many factors, including, among others, individual characteristics, including gender, but also institutional issues, such as regulatory policies, and the willingness of individuals to gamble and their risk aversion. Indeed, the decision to start gambling can be explained in the context of Badillo and López (2013)’s model for non-smokers starting to smoke, which is an extension of Kan (2007)’s model based on time inconsistent preferences. This approach overcomes the limitation of Becker and Murphy (1988)’s rational addiction model, which does not account for the possibility of different planned and actual decisions. Under this theoretical framework, depending on the value of the time inconsistency parameter, it is possible that, given the utility of starting to gamble in the current period, the utility of gambling and the future discount factor, a non-gambler individual will plan not gambling in the future but at the same time may decide to gamble in the current period (inconsistent preferences). In such a case, lowering the net benefits of starting gambling (e.g. through mechanisms which increase the cost of start gambling) will help to overcome this inconsistency. On the other hand, any action that makes it easier to start gambling (that is, lowering its cost) will maintain this inconsistency or make it more likely that the planned and the actual decision to start gambling are not at conflict with each other.

Accordingly, any gambling episode is ultimately determined not only by quite different factors that affect individual decision-making (that is, whether or not to participate and, if so, the optimal amount of spending) and behaviour, but also by an easier access to gambling itself (e.g. liberal gambling-related regulatory changes may encourage people to start gambling by lowering the costs). Thus, if consumers prefer a corner solution to these decisions (that is, they choose not to gamble), an expansion of gambling opportunities will pose no effect to consumer’s behaviour (Kearney, 2005).

As for gender, recent literature has commented on a lack of gender specific research, both into gambling in general and problem gambling in particular (2001; McCarthy et al., 2019). In general, the gambling experiences of women have been somewhat hidden amongst nationally representative statistics and long-held assumptions about preferred activities and less frequent participation (2001). Kairouz et al. (2023) conclude that there is a scarcity of socio-cultural studies of gender in gambling scholarship, indicating the need to expand sociocultural analysis in research on gender and gambling.

Overall, there is strong evidence of gender differences in gambling motivations (Wenzel & Dahl, 2009) and, generally, men and women have shown different levels of gambling involvement with men experiencing higher levels of engagement and prevalence than women (Stoltenberg et al., 2008). Indeed, men gambled more frequently and had higher losses and wins (Welte et al., 2004). However, in the last decade, some prevalence studies have shown that gambling participation rates are roughly similar for both women and men (McCarthy et al., 2019). Based on research, males may be more likely to gamble for excitement or thrill seeking, while for women, gambling may be related to modulation of adverse moods (McCormack et al., 2014).

Women are more likely to start gambling at an older age than men (Wenzel & Dahl, 2009), while men were found to be more likely than women to participate regularly in most forms of gambling (Svensson et al., 2011) and to gamble more frequently and with higher expenditure (Hing & Breen, 2001). Recently, it is shown that nearly half (42%) of women have gambled in the last four weeks, predominantly on activities such as the lottery, scratch cards and bingo. Women aged 35–54 are most likely to gamble (32%), with slightly lower participation amongst younger and older age groups. Lotteries and scratch cards are universally popular, but younger women are also found to gamble privately with their friends, and playing slot machines in gambling outlets. Women are also beginning to engage in online gambling products (Gambling Commission) and they seem to be more likely to be influenced by gambling advertisements (McCormack et al., 2014).

Also gender differences are observed about gambling risks. Men tend to be more sensation-seeking and risk-takers than women (Harris & Jenkins, 2006). Previous evidence indicated that 2.9% of women were problem gamblers compared to 4.2% of men (Wong et al., 2013). Latest prevalence data suggests that the problem gambling rate is 0.2% amongst women, with the moderate and low risk rates at 0.9% and 1.4% respectively. The problem gambling and low risk rates for women are both lower than male counterparts (Gambling Commission). This is consistent with previous studies (Hing et al., 2016) that found that significantly higher proportions of males scored as low risk, moderate risk and problem gamblers compared to females, suggesting gender-based differences in risk factors.

This paper is expected to address a deficiency of gender-specific research into gambling. It aims to analyse how individual factors reportedly associated with gambling consumption interact with the institutional setting, which is determined by the availability of gambling opportunities, in the propensity to start gambling, paying special attention to gender differences and to the duration dependence effect and the time effect, which might be associated to regulatory changes. Even with an increase in gambling opportunities for women (Welte et al., 2011) the question is whether gender differences in gambling engagement are still found. Particularly, it is expected to get a much better understanding of what motivates women to gamble and how they engage with different gambling products. That is, the focus will be on those factors that may affect individuals’ spell length as non-gamblers.

The case study comes from Spain, where the domestic gambling market has seen a dramatic increase in both economic figures and opportunities over the last decades. Until 1977, legal gambling in Spain was largely limited to football pools, the Spanish National Lottery (*Lotería Nacional*) and the charity lottery for the benefit of the visually impaired, with the exception of certain activities specifically sanctioned by the then-dictatorship government.

The arrival of democracy brought along its first licenses that further legalized additional gambling products. In fact, the re-established democratic government acknowledged that all prior absolute prohibitions systems had not only failed in their “moralizing efforts,” but had also encouraged a situation of “generalized clandestine gambling, with even more real risks” (Royal Decree-Law 16/1977, of 25 February). In order to provide legal certainty, and to meet the objectives of “social protection and guardianship” and “the defence and promotion of fiscal interests,” almost every gambling game and activity were mass legalized, including lotteries, betting, casinos, bingos, and slot machines. This resulted in a significant increase in both gambling participation and expenditure. In fact, the Spanish gambling market saw a dramatic expansion between 1977 and 1985 (Jiménez-Murcia et al., 2014), expenditure on gambling products increased by more than 350% (5,650 million euros in 1977 to 25,860 million euros in 1985; figures in 2000 real euros).

The next major expansion took place in 1985. *La Primitiva* was re-established after being eventually abolished in 1862, and it quickly became, and still remains as of this writing, the most popular lottery game marketed on a regular basis in Spain. A few years later, and thanks to the growing popularity of lottery-type games, lottery mania, as Kaplan (1990) calls it, spread across the country: *BonoLoto* was introduced in 1988, and *El Gordo de la Primitiva*, which was presented as a derivative of *La Primitiva* but with lower odds and higher jackpots, was launched in 1993. Gambling consolidated over the 1990s, as its share of the domestic GDP remained stable at around 4% between 1995 and 2000. Further developments continued well into the 21st century with the arrival of new games, such as the transnational lottery EuroMillions in 2004, of which Spain was a founding member.

Apart from the introduction of some new gambling products and regional lottery operators, as it was the case of *Lotería de Catalunya* (García & Pérez, 2022 examine competition between lottery agencies in the same jurisdiction in the case of Catalan lotteries), no major changes to the legislation were made until the 2011 Spanish gambling law (Law 13/2011, of 27 May) came into force, which addressed online gambling for the first time. Until then, online gambling was not strictly forbidden, but it lacked a specific regulation that would provide legal certainty to both operators and consumers. This new gambling law was the result of the fundamental changes that had occurred in gambling following its decriminalization in 1977 and its subsequent popularization over the decades, and the introduction of new Internet-enabled devices that facilitated access to a huge supply of online gambling activities. In fact, online gambling has become very relevant in the last few years, and the government has recently passed new restrictions targeting online outlets advertising in particular.

Currently, operating gambling activities in Spain requires obtaining permission from the appropriate authorities. It is the Directorate General for the Regulation of Gambling (Ministry of Consumer Affairs) that manages the regulation, licensing, supervision, coordination, control and sanctioning of gambling activities at state level, including online gambling, lottery products and football pools operated by the Spanish National Lottery Agency (SELAE) and those managed by the Spanish National Organization of the Blind (ONCE).

Since the regulatory and fiscal authority over privately operated gambling in Spain (casinos, bingos, slots, betting bookmaker, etc.) were transferred to the regional governments, offline gambling opportunities at regional level differ significantly and are subject to approval by each regional government. As an instrument of coordination, a Gambling Policy Council with representatives of both the central government and regional governments (autonomous communities) has been established.

As previously hypothesized, all of the aforementioned regulatory and institutional changes related to gambling in the period under consideration are expected to make gambling easier by reducing the costs of getting started gambling, due to either the expansion of gambling opportunities or the allowance of Internet gambling. Of course easier access to any product or service will increase the likelihood of people consuming it, and this will hold true for gambling. While it is obvious that easier access will lead to people being more likely to gamble, it would be interesting to determine whether different ways in which that access is increased will have a stronger effect than others.

Simultaneously, individuals’ socio-demographics are expected to have an impact on gambling decision making due to the heterogeneity associated with the utilities of different states. Finally, gender differences may play a role in consumers’ behavioural reactions to changes in market regulations and supply opportunities. In fact, data analysed by NatCen and the University of Liverpool suggests that women who have online gambling accounts, actually tend to play more often, for longer, and spend more than men (Forrest & McHale, 2021).

Using data from the “Study on prevalence, behavior and characteristics of gambling users in Spain” (Dirección General de Ordenación del Juego, 2015), this paper deals with the propensity of individuals to start gambling, by analyzing what factors might, in fact, lead individuals to eventually engage in gambling activities in a time changing regulatory environment and whether they have a different effect on women and men. It is known that online gambling products are often enjoyed by people who want to have some leisure time and relax, however, there are lots of different reasons why people gamble (Binde, 2013). For many women, gambling provides an opportunity to be sociable and enjoy time with friends, with activities such as gambling at casinos, going to the bingo and playing slot machines in gambling outlets providing the opportunity to gamble whilst having fun with others (2001).

To fully consider these individuals who are inherently non-gamblers, here we propose a split population duration model to accommodate this empirical research. This methodology has been proved successful in studying the propensity to start and quit consuming other potentially addictive products, like tobacco and alcohol use (e.g., Douglas and Hariharan, 1994; Forster & Jones, 2001; López-Nicolás, 2002). Nevertheless, to the best of our knowledge, there are no similar studies on this issue. A better understanding of the characteristics of individuals who eventually decide to gamble, specially what is the difference (if any) between how women gamble and how men gamble, and the potential consequences of the expansion of gambling opportunities will be helpful for policy makers and gambling stakeholders.

## Data and Variables

The Spanish prevalence survey provides a nationally representative dataset of the Spanish population, consisting of 6,816 individuals aged 18 and over, as the legal age for gambling, who answered to a personal survey regarding sociodemographic and gambling factors. The data was gathered by means of personal interviews. Individuals who failed to respond to key variables (which makes it impossible to determine how long individuals have been gambling for, and therefore, the subsequent survival analysis) were removed from the sample. Overall, 860 individuals were dismissed (12.61% of the sample), most of whom (828) did not remember their age when they started gambling. Consequently, the final sample consisted of 5,956 individuals - about half (52%) of women (52.6% in the original study) -. Resulting participation ratios (gambling at least once) were very similar to those provided by the original prevalence survey (75.8% and 75.7%, respectively). In addition, the weights of the original data were recalculated to take into account the characteristics of the final sample.

The survey did not provide any information about the spell length as a non-gambler. However, the spell length (in years) was inferred based on available information on the exact age, starting age and gambling status of respondents. The duration of current self-declared gamblers as non-gamblers was established as the number of years elapsed until they first gambled minus 13, while the duration of current self-declared non-gamblers was determined to be their exact age at the time of the survey minus 13, for their spells as non-gamblers had not yet ended at the time of the survey. We subtracted 13 years from each calculation because descriptive statistics showed that a significant proportion of the individuals started gambling at 14, whereas it was anecdotal at earlier ages. It should be noted, though, that while the legal gambling age in Spain is 18, gambling is still easily accessible to minors.

To analyse the propensity to start gambling, exogenous factors that are believed to affect individuals’ attitudes towards gambling and, therefore, to have an impact on the hazards of starting gambling were also included. Gender and education level are common covariates in gambling research, for gambling patterns and behaviour are strongly determined by socio-demographic conditions (see Layton and Worthington, 1999; Wardle et al., 2011; Welte et al., 2002, and Worthington, 2001, among others, for further insight). Existing literature has not found consistent evidence of how personal income affects gambling participation; however, we decided to include the real GDP per capita growth rate as a proxy for the variation of disposable income over the last century, as it seemed reasonable that the remarkable growth of the GDP might have had some impact on the likelihood of ending the spell as a non-gambler.

In addition, time effects on the probability of starting gambling, linked to changes in regulation in the Spanish gambling industry, are expected to be caught by a set of dummy variables defined for each of the following periods: from 1977 (first ever legalization of privately owned and operated gambling) through 1985 (introduction of the first lotto-type games), from 1986 to 2010 (a period in which lotto markets and offline gambling activities expanded in a regular manner over the years), and 2011 (first ever specific regulation of online gambling) onwards. The period prior to 1977, when legal gambling was severely restricted and non-legal gambling mostly criminalized, remained as the reference category.

Descriptive statistics reported in Table 1 show that about 73% of respondents have gambled at least once in their lifetime, most of whom (about 90%) did so in the previous year (that is, 2014). For those who self-identified as gamblers, their average starting age was about 23 years, whereas their average spell duration as non-gamblers was about 10 years. As mentioned above, notice that the reported minimum starting age was 14 years old. Indeed, gambling is only legal for adults, but this does not prevent younger people from accessing some of the gambling modalities covered by the prevalence study.

**Table 1:** Summary statistics

*Variable*	*Mean*	*SD*	*Min.*	*Max.*
**Gambling at least once** (Yes = 1)	0.758	0.429	0	1
**Starting age**	22.85	8.15	14	75
**Age**	47.01	17.58	18	95
**Gender** (Men = 1)	0.483	0.500	0	1
**Education level**				
*No education*	0.058	0.233	0	1
*Primary*	0.286	0.452	0	1
*Secondary*	0.423	0.494	0	1
*Higher*	0.233	0.423	0	1

*Notes: Descriptive statistics were calculated using weighted data*

The average age of respondents was 47 years old. Gender was evenly distributed, but over 65% of individuals had at least a secondary degree (high school or higher).

## Econometric Modelling

As we mentioned in the introduction, the goal of this research is to assess the factors that could influence the propensity to start gambling. This propensity can be understood in terms of the probability of a variable associated with the length of the spell as a non-gambler being equal to a particular value (*t*) conditional on being a non-gambler for *t* periods. This means that this propensity can be expressed in terms of the distribution of a positive random variable, i.e., a duration variable, which measures for how long an individual is in a particular state, in this case being a non-gambler. As far as we known, only Forrest and McHale (2016) and Kainulainen (2021) have used survival analysis to study between-session loss-chasing behaviour by loyalty card holders playing slot machines and how past gambling outcomes affect current gambling consumption, respectively.

The data set from the prevalence survey is actually a retrospective cross-section survey in which individuals self-reported whether or not they were gamblers, and, if so, the age at which they started gambling. This means that the sample consists of complete (uncensored) spells corresponding to individuals who had already started gambling at the time of the interview (*d*_*i*_ = 1) and right-censored spells corresponding to individuals who had not yet (*d*_*i*_ = 0). The latter, specifically referred to as incomplete spells, are in fact formed from two different types of individuals: those who are inherently non-gamblers and, therefore, will never gamble, and those who would eventually have been observed gambling had the monitoring been longer. The survey did not include any information to distinguish the two.

The specific characteristics of the data are relevant to the duration analysis, as they determine how the duration models have to be specified and estimated (Allison, 1982). In this regard, since we have both complete and incomplete spells, standard duration models, which only consider complete spells, are not suitable for this data.

On the other hand, previous econometric research (Douglas & Hariharan, 1994; Forster & Jones, 2001; López-Nicolás, 2002; Schmidt and Witte, 1989) has, however, developed what it is now known as split population duration models to deal with cases like the one we face here in which two potential types of individuals can be associated with those who are still in a particular state (non-gambler) when interviewed (incomplete spells). The two types are defined depending on whether they will abandon the state (start gambling) or not (never gambling) in the future. The specification of this split population model considers two equations: first, a discrete choice model to estimate whether or not individuals will eventually start gambling in the future, and second, the proper duration model to estimate the spell length as non-gamblers.

Additionally, how the duration variable is measured, in this case in years, must be taken into account (Allison, 1982). Here, we use a discrete-time duration model to avoid potential biases associated with continuous-time models. This approach is more flexible in specifying the duration dependence patterns of the conditional propensity to start gambling.

The econometric specification is as follows. Let ${s}_{i}$be a dummy variable set to 1 for an individual $i$ who will eventually start gambling and 0 otherwise, for which the following binary discrete-choice logit model is defined:

1 $$Prob\left({s}_{i}=1\right)=F\left({Z}_{i}^{{\prime }}\delta \right)$$

2 $$Prob\left({s}_{i}=0\right)=1-F\left({Z}_{i}^{{\prime }}\delta \right)$$

where $F$ is the cumulative distribution function of a logistic distribution,${Z}_{i}^{{\prime }}$ is the covariates vector and $\delta$ is the parameters vector. Notice that *s*_*i*_ is not observed.

On one hand, the contribution to the log-likelihood function of individuals who have already started gambling (*d*_*i*_ = 1) at period ${t}_{i}$ is the probability of eventually being a gambler (*s*_*i*_ = 1) times the probability of starting gambling at period ${t}_{i}$ (that is, $Prob\left({T}_{i}={t}_{i}\right)$, where ${T}_{i}$ is the random variable of the spell length as a non-gambler). On the other hand, the contribution of individuals who are not observed to start gambling (*d*_*i*_ = 0) is the probability of never starting gambling (*s*_*i*_ = 0) plus the probability of starting gambling after ${t}_{i}$ (that is, $Prob\left({T}_{i}>{t}_{i}\right)$, which actually is the survivor function). The resulting likelihood function is the following:

3 $$\text{l}\text{o}\text{g} \text{L}=\sum _{i=1}^{N}{d}_{i}\text{log}\left[Prob\left({s}_{i}=1\right) Prob\left({T}_{i}={t}_{i}\right)\right]+\left(1-{d}_{i}\right)\text{log}[1-Prob\left({s}_{i}=1\right)+Prob\left({s}_{i}=1\right) Prob\left({T}_{i}>{t}_{i}\right)]$$

If we define the discrete time hazard rate as ${h}_{i{t}_{i}}$ as the probability of the *ith* individual starting gambling in period *t*_*i*_ conditional on being a non-gambler for *t*_*i*_ periods ${[h}_{i{t}_{i}}=Prob({T}_{i}={t}_{i}|{T}_{i}\ge {t}_{i}\left)\right]$, then:

4 $$Prob\left({T}_{i}={t}_{i}\right)={h}_{i{t}_{i}}\prod _{k=1}^{{t}_{i}-1}(1-{h}_{ik})$$

Notice that there is a one-to-one relationship between the hazard function and the survivor function. It is also relevant to point out that the basic contributions in the duration model $Prob\left({T}_{i}={t}_{i}\right)$ and $Prob\left({T}_{i}>{t}_{i}\right)$ are the result of evaluating the probabilities of starting gambling (or not) in each period, conditional on being a non-gambler, ${h}_{i{t}_{i}}$ and ${1-h}_{i{t}_{i}}$, respectively, according to a binary discrete choice model.

We chose a logistic specification for ${h}_{i{t}_{i}}$, i.e.:

5 $${h}_{it}=\frac{exp\left[\theta \left(t\right)+{X}_{it}^{{\prime }}\beta \right]}{1+exp\left[\theta \left(t\right)+{X}_{it}^{{\prime }}\beta \right]}$$

where $\theta \left(t\right)$ is a 4th order polynomial function of $t$ to capture the potential duration dependence of the hazard rate, ${X}_{it}$ is the covariates vector (including time-varying factors and individuals’ personal characteristics such as gender) and *β* is the vector of parameters. In particular, we pay specific attention to the effect of gender in the conditional probability ${h}_{i{t}_{i}}$ and how it evolved through time. The polynomial function accommodates a flexible specification of the duration dependence of the hazard rate without affecting the significance of estimated coefficients. Also, the fit of this model is better than that of either lower order polynomials or those using a complementary log-log specification for the hazard function instead of the logistic function used here.

## Results

Table 2 shows the estimated coefficients for the propensity to starting gambling for both the split population model and the standard model with no participation equation (all uncompleted spells are considered as censored). The split population model provides a better fit in terms of the value of the Akaike Information Criterion (AIC). Overall, estimates in both models were strongly statistically significant.

**Table 2:** Coefficient estimates for duration models

	*Split population*	*Standard model*
**Participation equation**		
Age cohort	-0.009 ^**^	
Education (1 = No studies)		
*Primary education*	1.020 ^***^	
*Secondary education*	0.898 ^***^	
*Higher education*	0.890 ^***^	
Constant	1.212 ^***^	
**Hazard function**		
Gender (1 = male)	0.888 ^***^	0.744 ^***^
GDPpc	-0.010 ^*^	-0.001
Regulation period (1 = before 1977)		
*1977–1985*	0.694 ^***^	0.816 ^***^
*1985 and after*	0.903 ^***^	0.944 ^***^
*2011 and after*	1.114 ^***^	1.153^***^
Gender * (1977–1985)	-0.317 ^***^	-0.372 ^***^
Gender * (1985 and after)	-0.199 ^**^	-0.363 ^***^
Gender * (2011 and after)	-0.330 ^**^	-0.693 ^***^
Trend	0.405 ^***^	0.384 ^***^
Trend^2^	-0.030 ^***^	-0.033^***^
Trend^3^/1000	0.782 ^***^	0.860 ^***^
Trend^4^/10^6^	-0.667 ^***^	-0.740 ^***^
Constant	-4.605 ^***^	-4.637 ^***^
log L	-20093.5	-20226.8
AIC	40161.0	40427.6

Notes: *** Significance at 1%; ** significance at 5%; * significance at 10%

Although it is aimed to assess gender differences on the propensity to start gambling, results of the control variables included in Table 2 are also discussed.

The age of the individual when interviewed is controlling for a cohort effect. In this regard, younger generations have a higher conditional probability of starting gambling, as shown in Table 3. Notice that the size of the effect of age is different depending on the educational level.

**Table 3:** Estimated probabilities (%) of starting to gamble

	*Education level*			
*Age*	*No education*	*Primary*	*Secondary*	*University*
**20**	73.80	88.65	87.36	87.27
**30**	72.06	87.73	86.36	86.26
**40**	70.25	86.75	85.29	85.18
**50**	68.38	85.71	84.15	84.04
**60**	66.45	84.59	82.94	82.82

A negative relationship between education and gambling could be anticipated (Coups et al., 1998), but the education level was actually found to have a positive effect on the propensity to gamble. Those without studies are less likely to start gambling than those with a higher educational level, as shown in Table 3. In fact, recent studies suggest that, contrary to what might be expected, people with higher IQ are more likely to spend more on, and be more successful in, certain gambling activities (Muela et al., 2021; Suhonen et al., 2020). Also, Humphreys and Perez (2012) found that Internet gamblers in UK report relatively high levels of education, in terms of the fraction that have college degrees.

Coefficients of the duration model showed that the real GDP per capita growth rate had a negative effect on the propensity to start gambling, although weakly significant (10% significance level). In fact, there is no significant effect in the standard duration model. This is not surprising at all, as previous research observed quite contradictory findings on this relationship. Kaizeler and Faustino (2011) found an upside-down U-shape relationship between gambling per capita (lottery sales) and GDP per capita, while Gandullia and Leporatti (2018) suggested that regional economic conditions (measured in GDP per capita) had differently affected diverse gambling products and categories.

Focusing on the gender coefficient, it was reportedly positive, hinting that men had shorter spell lengths than women (that is, men started gambling earlier than women), but its interactions with the gambling regime time period dummies suggested that this gap in the spell length has been gradually narrowing with each successive change in gambling regulation. However, men are well known to be less risk-averse and more susceptible to over-confidence (Chalmers & Willoughby, 2006) and tend to play more often and spend more than women on gambling products (Kitchen & Powells, 1991; Sawkins & Dickie, 2002; Welte et al., 2002). All of this may well condition their behaviour and make them more likely to gamble.

**Fig. 1:**
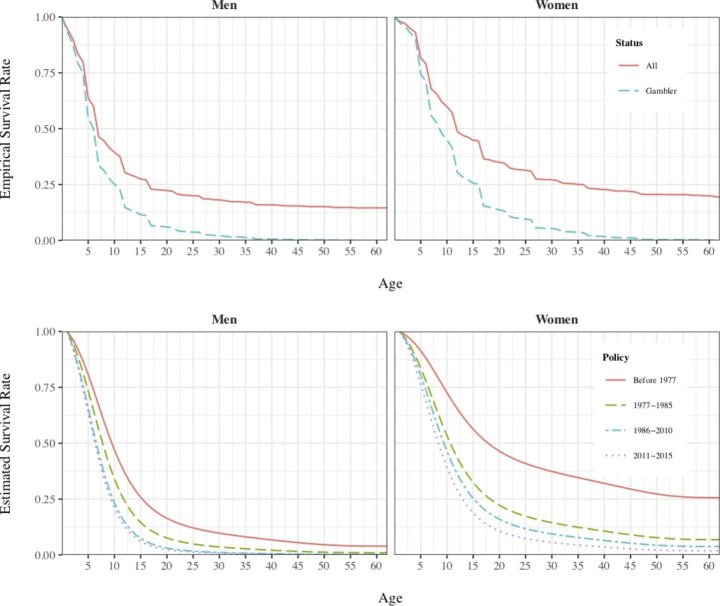
Comparison of Survival Functions (Empirical and Estimated)

Estimated coefficients for time period dummies linked to changes in gambling regulation were also positive. Defining the period prior to 1977 (when gambling was mostly banned in the country) as the reference period, each new time period corresponding to major changes in gambling legislation is positive correlated with the propensity to start gambling for both men and women, shortening the spell further than the previous one. However, interaction terms suggest that women’s spells have shortened more with every new period than men’s. These hints become quite evident in Fig. 1, which plots the empirical and the estimated survival function for starting gambling for both men and women and for each considered regulation period.

Estimated survival functions, which plot the probability of staying as a non-gambler after *t* periods, shifted to the left with each new legislation, meaning that each change in gambling regulation is linked to a short in the spell length as a non-gambler. For men, 75% of them took 15 years to start gambling (note that the spell starts at age 14, as gambling was anecdotal at earlier ages; we discussed this in the descriptive statistics section) in 1977, whereas it now takes less than 10 years after the 2011 reform. It was worse for women: the survival function never reached the 75% mark in 1977, as it became asymptotic, but now only takes 12.5 years. Furthermore, survival functions decreased more rapidly for females than for males, as observed in the estimated coefficients (Table 2). Gambling patterns have clearly changed since 1977, which is not surprising, because gambling was mostly banned before that year. Of course, people were expected to turn more likely to gamble when it became legal and widely available.

When considering only gender differences, men were still more likely to start gambling at earlier ages. However, survival rates decreased more for women than for men. Differences between males and females’ patterns are much more evident before 1977 than in more recent periods.

Note that the empirical survival functions for both males and females do not converge to zero when considering the whole sample due to incomplete spells from non-gamblers, while the estimated survival functions do converge to zero for males mainly due to the pattern prior to 1977.

**Fig. 2:**
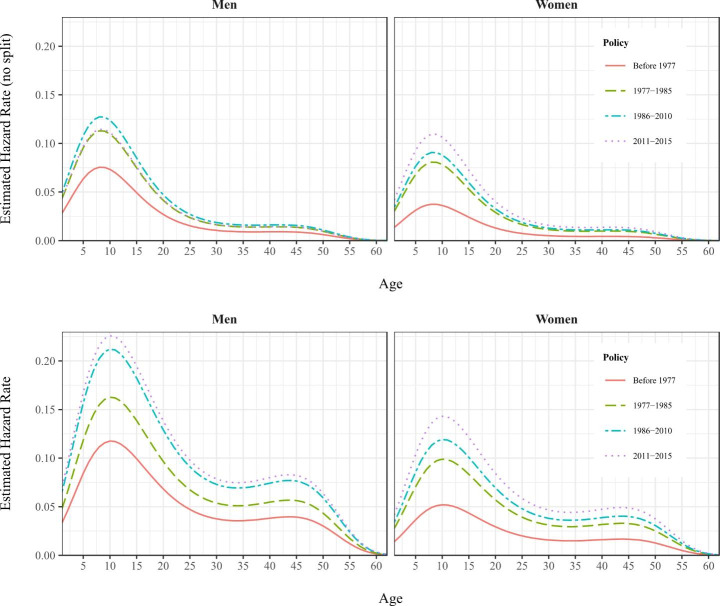
Comparison of Hazard Functions (No Split and Split Population)

The propensity to start gambling of Fig. 2, defined as one’s probability to start gambling conditional on one’s survival as a non-gambler up to time *t*, also pointed in the same direction. Time effects between 1977 and 2011 are connected with successively increases in the hazard of starting gambling: the risk is now two times higher for men and three times higher for women than before.

Overall, the probability of starting gambling increased more for women (in both absolute and relative terms), although it was still lower than men’s. Both genders followed the same trend. Risk increased roughly up to the first 10 years of survival, meaning that the period with the highest risk of starting gambling is around 24 years old. From then, the risk gradually decreased over time until the 35-years mark, at which point it remained quite constant for several years. It then decreased again to zero at around the 60-years mark.

It is also worth noting that the estimated hazard rates have substantial differences in size—not as much in the pattern they follow—when the split population model, which do consider incomplete spells from non-gamblers, is used instead of the standard one.

## Concluding Remarks

This paper offered a main contribution to the public debate regarding the consequences of an expansion of gambling opportunities. Based on a time inconsistent preferences theoretical framework, we use a specific duration model, which is a rather novel approach for gambling research, to provide an empirical analysis of how individuals’ propensity to start gambling responded to gender differences and other individual characteristics (age, education), but also to time effects, which might be associated to gambling regulatory changes over time. Duration models are appropriate for measuring and analysing the length of time that individuals spend in a given state before transitioning to a different state.

The data set used came from a cross-section retrospective survey provided by the Directorate General for the Regulation of Gambling of Spain. This survey was one piece of the Directorate’s 2015 comprehensive “study on the prevalence, behaviour and characteristics of gamblers in Spain”. We specifically proposed a split population duration model to accommodate both complete and incomplete (right-censored) spells coming from non-gamblers. This split population model included time-varying regressors and individuals’ personal characteristics (including gender). The main objective was to assess the effect of gender on the propensity to start gambling, putting particular attention to time effects linked to different regulatory periods.

Overall, expanding gambling opportunities and availability (that is, what makes gambling easier by lowering its costs) is positive correlated with people being more likely to start gambling. However, different ways in which that access was increased have had a stronger effect than others. This is a quite important finding in terms of policy implications since public health concerns over gambling issues have been the strongest argument against the widespread expansion of gambling opportunities. In particular, gender was found to have a significant effect on the propensity to start gambling, suggesting that men were more likely to gamble than women. Furthermore, the interactions between gender and time effects dummies showed men were still more prone to participate in gambling activities through time, but women’s spell length as non-gamblers decreased more over time. The propensity to start gambling was maximized at about 24 years old; it first increased with age, and then decreased to zero over time. Currently, both men and women are clearly more likely to start gambling at earlier ages than before. However, this is not unexpected, because gambling was mostly banned prior to 1977, meaning that of course gambling patterns would change over time. In any case, a better understand of gender differences in terms of consumer decision making about gambling could be helpful for decision makers and gambling regulators and stakeholders.

As for evidence-based regulators this paper results may significantly enhance the ability to understand gambling behaviours amongst subgroups of the population, including women, and to enable to better identify ways to improve gambling regulations.

Notwithstanding, some limitations to this research should be noted. Cross-sectional studies such as this one do not allow for a full understanding of the underlying process. Research would benefit from longitudinal studies that provide data on changes in individuals’ personal factors over time and a more detailed information of the supply of gambling products.

## Data Availability

Data associated with the study is publicly available to download at https://www.ordenacionjuego.es/es/estudio-prevalencia.
